# Supplementary material for: Drone swarm strategy for the detection and tracking of occluded targets in complex environments
Source: Commun Eng. 2023 Aug 2;2:55. doi: 10.1038/s44172-023-00104-0 (PMC10956045; doi:10.1038/s44172-023-00104-0)
Supplement: Supplementary file 2 — Supplementary Information [file 44172_2023_104_MOESM2_ESM.pdf]

Supplementary Information

Supplementary Note 1: Simulated vs. Real Integrals

Figure S1 illustrates the difference between integral images that are simulated for our procedural forest and integral images that are captured with a physical drone over real mixed forest. Both show an upright standing person (blue cloth, black hair). Note that sensor errors, such as GPS imprecision or camera noise, are not simulated and that the cloth color of the avatar in the simulation was only roughly matched with the cloth color of the real person. In contrast to the the simulations, real integrals suffer from artifacts and blur that are due to misregistrations caused by GPS errors and camera noise. This example, however, indicates that simulated integrals approximate real integrals well, and that our simulations are not too far from real-world conditions.

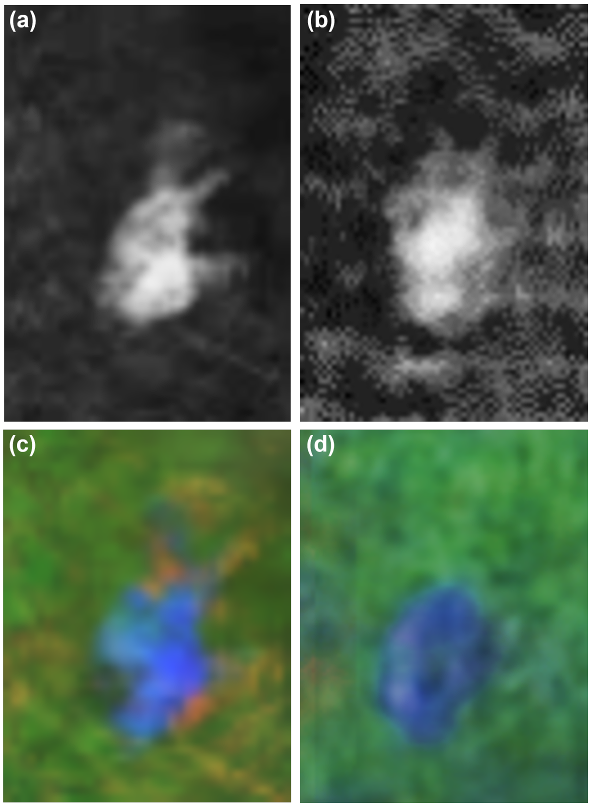

Fig. S1 Simulated vs. Real integrals. Simulated integral images for our procedural forest (**a**: thermal, **c**: RGB) versus integral images captured with a physical camera drone above real mixed forest (**b**: thermal, **d**: RGB). The close-ups are scaled to the same dimensions on the forest ground.

Supplementary Note 2: Different vs. Same Altitude

Figure S2 visualizes the difference of simulated integral images computed for drones with varying altitudes (as explained in Collision Avoidance) versus integral images that are computed for drones with the same altitude. Note that focal length is considered in the simulation but sensor errors, such as GPS imprecision, lens distortion or camera noise, are not simulated. They would add additional blur and noise in both cases, which would make the difference between the two cases even less. Note also that minor difference are also due to perspective changes caused by the different altitudes, while spatial sampling remains mainly unaffected.

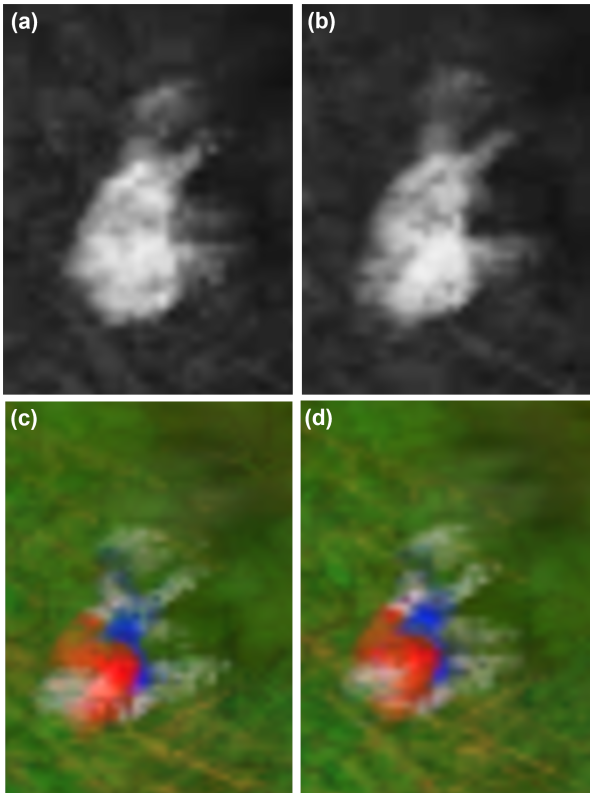

Fig. S2 Different vs. Same altitude. Simulated integral images computed for drones with various altitudes (**a**: thermal, **c**: RGB, altitude range: 35-44 m at 1 m steps) versus simulated integral images computed for drones with the same (average of 35-44 m) altitude (**b**: thermal, **d**: RGB, altitude: 40 m). Note that the lateral positions of the ($n = 10$) drones are identical in both cases.

Supplementary Note 3: Impact of Swarm Size and Forest Density on Target Visibility

Full simulation runs of the results shown in Figure 4 are presented in Figure S3 (i & ii). Note, that the simulation duration is predefined while the PSO stops if no further progress in the objective function is achieved (indicated by the dots). Furthermore, Figs. S3 (iii & iv) present averaged results of three simulation runs for each of the six scenarios shown in Figure 4. Despite the randomness of the forests and in the PSO algorithm, all of these runs reveal a consistent behaviour.

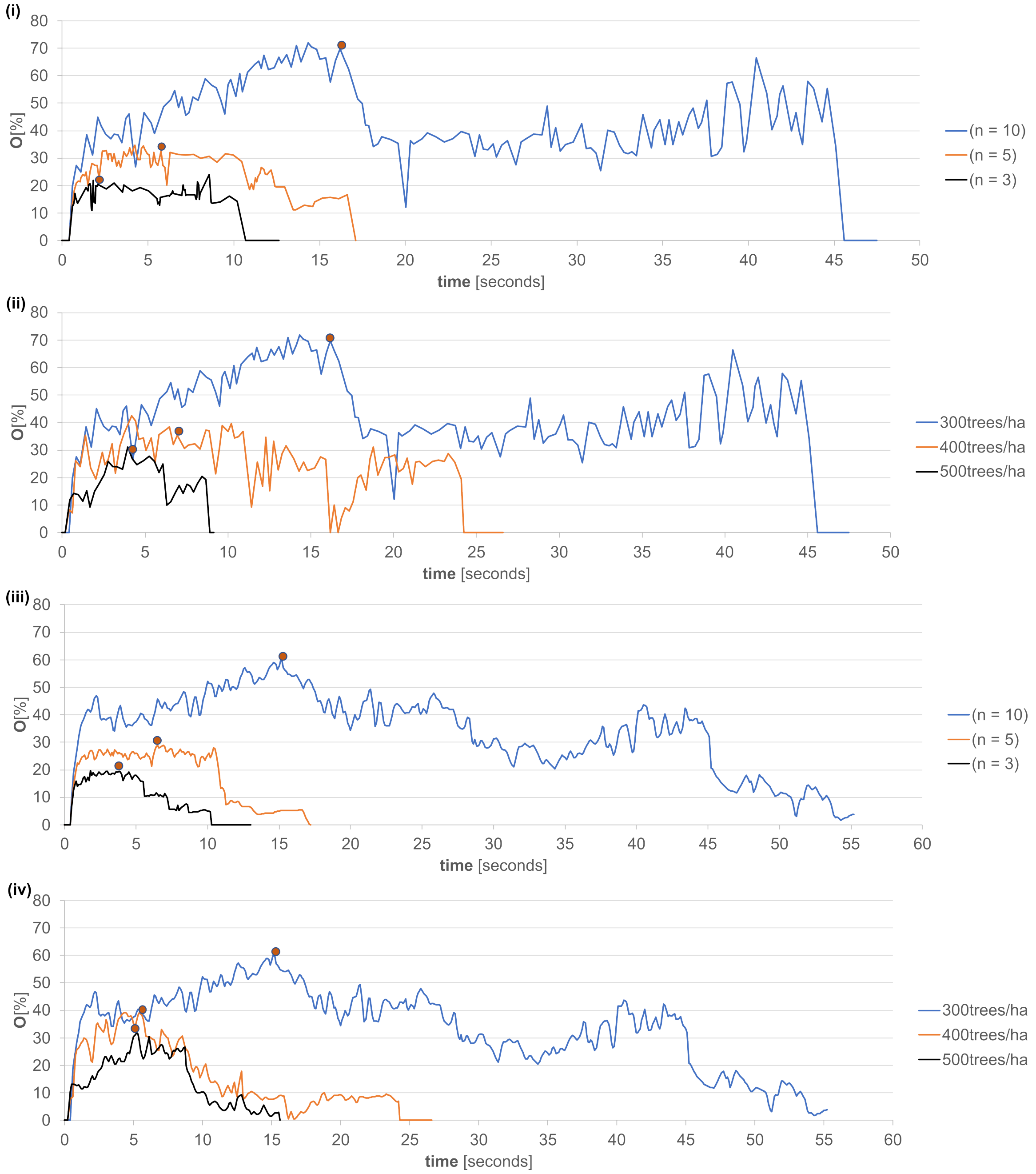

Fig. S3 Impact of swarm size and forest density on target visibility. (**i** & **iii**) Increasing swarm size (n = 3,5,10) leads to better target visibility. Here, a wider synthetic aperture and a larger number of samples increases the target visibility and consequently the probability of its detection with a constant forest density $(300\ trees/ha)$. (**ii** & **iv**) Increasing forest density $(300, 400, 500\ trees/ha)$ decreases target visibility due to denser occlusion. Here, the swarm size is constant $(n = 10)$. The plots show full and (**iii** & **iv**) averaged simulation runs of the results presented in Figure 4.

Supplementary Note 4: Another Motion Example

In Fig. S4, another motion example is illustrated. Here, the target moves on a circular path, without stopping. Although the swarm loses it due to too dense occlusions at the bottom-left and bottom-right parts of the path, it is able re-detect and track it till the end. The average deviation between the target's ground truth position, motion speed, motion direction and the corresponding estimations of our PSO was 0.53 m, 0.088 m/s, and 10.35°, respectively.

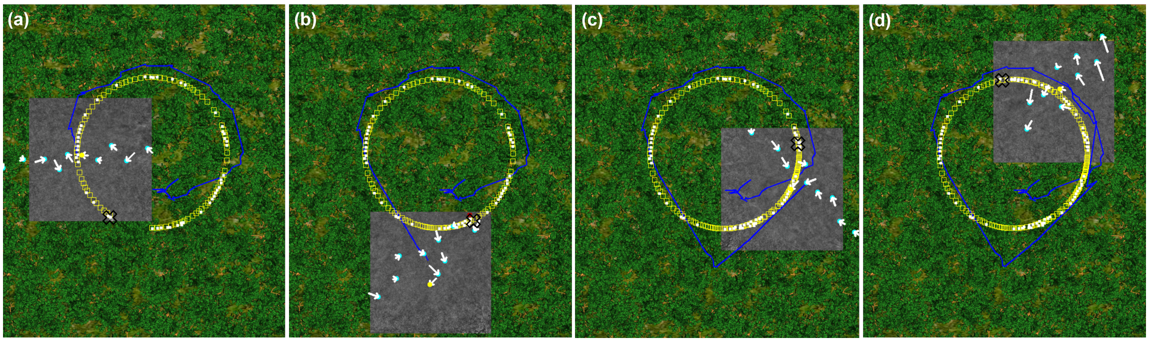

Fig. S4 Another motion example. Tracking a person moving on a circular path at constant speed (4 m/s): **(a)** The target is lost after 29.71 seconds, **(b)** re-detected after 32.32 seconds, lost again after 34.88 seconds, **(c)** re-detected after 39.628 seconds, and **(d)** tracked for the remaining 8.17 seconds. Note that the yellow boxes highlight the target's ground truth positions, the white stars indicate the target's estimated positions, the white arrows show the movement of the drones between time steps $t-1$ and t, the blue lines represent the total sampling paths of the swarm's center of gravity, the yellow dots indicate the the best sampling position at time t, the grey area illustrates the integrated ground coverage at time t, and the black crosses indicate the target's position at the presented simulation times. Simulation parameters (see Methods for details): drones' ground speed = $10\ m/s$, Δ_h = $1\ m$, forest density = $300\ trees/ha$, n = 10, T = 20.4%, h_1 = $35\ m$, c_1 = $1\ m$, c_2 = $2\ m$, c_3 = $1.953\ m$, $s = c_4$ = $4.2\ m$, c_5 = 0.3. See Supplementary Movie 5.

Supplementary Note 5: Failure Case - Too Fast Moving Target Tracking

The illustrated failure case in Figure S5 depicts a scenario where a too fast-moving target follows an uninterrupted circular path. Initially, the moving swarm is able to detect and track the target effectively. However, over time the high speed of the target causes it to eventually move beyond the range of the swarm's field of view, resulting in the swarm losing track of the target.

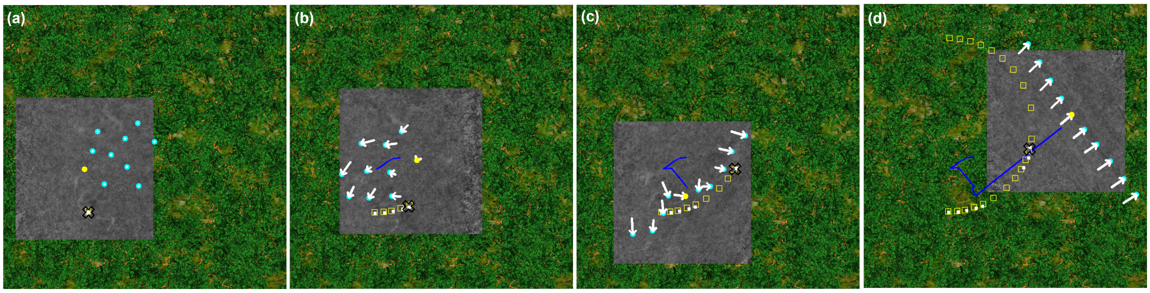

Fig. S5 Failure case - too fast moving target tracking. **(a)** Tracking a target moving on a circular path at constant speed (8 m/s): **(b)** The target is lost after 1.06 seconds, **(c)** re-detected after 2.591 seconds, **(d)** lost again after 3.176 seconds, and remains undetected for the remaining 5.51 seconds. Note that the yellow boxes highlight the target's ground truth positions, the white stars indicate the target's estimated positions, the white arrows show the movement of the drones between time steps $t-1$ and t, the blue lines represent the total sampling paths of the swarm's center of gravity, the yellow dots indicate the the best sampling position at time t, the grey area illustrates the integrated ground coverage at time t, and the black crosses indicate the target's position at the presented simulation times. Simulation parameters (see Methods for details): drones' ground speed = $10\ m/s$, Δ_h = $1\ m$, forest density = $300\ trees/ha$, n = 10, T = 23.3%, h_1 = $35\ m$, c_1 = $1\ m$, c_2 = $2\ m$, c_3 = $2.97\ m$, $s = c_4$ = $4.2\ m$, c_5 = 0.3. See Supplementary Movie 6.

Supplementary Note 6: Failure Case - Target Tracking in Too Densely Occluded Regions

The scenario depicted in Fig. S6 involves a target moving along a circular path, traversing from a sparse region to a too densely occluded region. The swarm detects and tracks the target efficiently in less dense regions. However, as the target moves towards a a densely occluded region and lingers there for an extended period, the swarm loses its ability to re-detect the target as it moves along the direction of the last detected target.

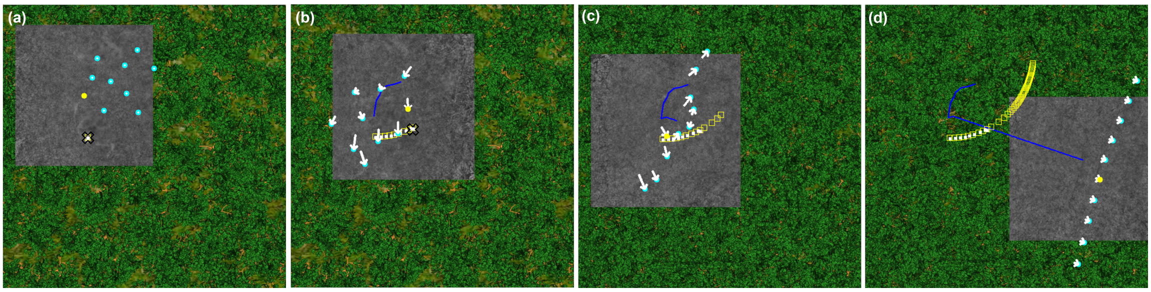

Fig. S6 Failure case - target tracking in too densely occluded regions. **(a)** Tracking a person moving at a constant speed (4 m/s) on a circular path: **(b)**The target is lost after 2.31 seconds, **(c)** as it moves in too densely occluded region, and **(d)** remains undetected for the remaining 5.07 seconds. Note that the yellow boxes highlight the target's ground truth positions, the white stars indicate the target's estimated positions, the white arrows show the movement of the drones between time steps $t-1$ and t, the blue lines represent the total sampling paths of the swarm's center of gravity, the yellow dots indicate the the best sampling position at time t, the grey area illustrates the integrated ground coverage at time t, and the black crosses indicate the target's position at the presented simulation times. Simulation parameters (see Methods for details): drones' ground speed = $10\,m/s$, Δ_h = $1\,m$, forest density = $300\,trees/ha$ (a-b) and $500\,trees/ha$ (c-d), n = 10, T = 16.8%, h_1 = $35\,m$, c_1 = $1\,m$, c_2 = $2\,m$, c_3 = $2.97\,m$, $s = c_4$ = $4.2\,m$, c_5 = 0.3. See Supplementary Movie 7.

Supplementary Note 7: Visibility Probability for Parallel-Sequential Sampling in Presence of Motion

Here we present the derivation of the visibility in an integral image X (as defined in [78]) of a hypothetical occlusion-free reference target signal S of size S_l moving at speed S_v.

The integral image X is the average of total N single images, where $N = N_p \cdot N_s$ (N_s sequential instances, being Δt apart from each other, of N_p images recorded in parallel at any instance t). Compared to our previous model, where the target signal S was static, a moving target signal S can now be present at any location in only N_o instances (where $N_o = \frac{S_l}{(S_v \cdot \Delta t)}$) and N_v ($N_v = N_p \cdot \mathrm{Min}(N_o, N_s)$) images.

Out of N image recordings, each single pixel pertaining to the target signal can be either occlusion free or occluded in only N_v images. We model this by the random variables: S (occlusion free), O (occluded), Z (determines if occluded or not), and D (occlusion probability). In N_v single image recordings, a pixel is D likely occluded ($Z_i = 1$). In this case the pixel's content is O_i. Otherwise it is $(1 - D)$ likely occlusion free ($Z_i = 0$). In this case it contains S:

$$X = \frac{1}{N}\left(\sum_{i=1}^{N_v}\left(Z_i O_i + (1 - Z_i)\, S\right) + \sum_{i=N_v+1}^{N} O_i\right). \tag{S1}$$

All random variables are independent and identically distributed with Z_i, following a Bernoulli distribution with success parameter D (i.e., $\mathrm{E}[Z_i] = \mathrm{E}[{Z_i}^2] = D$; furthermore, note that $\mathrm{E}[Z_i(1 - Z_i)] = 0$ is true). The random variable S follows a distribution with mean $\mathrm{E}[S] = \mu_s$ and $\mathrm{E}[S^2] = (\mu_s^2 + \sigma_s^2)$ and analogously O_i follows a distribution with mean $\mathrm{E}[O_i] = \mu_o$ and $\mathrm{E}[{O_i}^2] = (\mu_o^2 + \sigma_o^2)$.

From [78], visibility is given by

$$V = 1 - \mathrm{MSE}, \tag{S2}$$

where

$$\mathrm{MSE} = \mathrm{E}[(X - S)^2] = \mathrm{E}[X^2] - 2\,\mathrm{E}[XS] + \mathrm{E}[S^2]. \tag{S3}$$

Thus the first term of Eqn. S3 expands to

$$\mathrm{E}[X^2] = \mathrm{E}\left[\left(\frac{1}{N}\left(\sum_{i=1}^{N_v} Z_i O_i + (1 - Z_i)\,S + \sum_{i=N_v+1}^{N} O_i\right)\right)^2\right], \tag{S4}$$

$$\mathrm{E}[X^2] = \frac{1}{N^2}\,\mathrm{E}\left[\left(\sum_{i=1}^{N_v} Z_i O_i + (1 - Z_i)\,S + \sum_{i=N_v+1}^{N} O_i\right)\left(\sum_{k=1}^{N_v} Z_k O_k + (1 - Z_k)\,S + \sum_{k=N_v+1}^{N} O_k\right)\right]. \tag{S5}$$

By distributive law,

$$\begin{aligned}\mathrm{E}[X^2] = \frac{1}{N^2}\,\mathrm{E}\Bigg[&\left(\sum_{i=1}^{N_v} Z_i O_i + (1 - Z_i)\,S\right)\left(\sum_{k=1}^{N_v} Z_k O_k + (1 - Z_k)\,S\right)\\ &+ \left(\sum_{i=1}^{N_v} Z_i O_i + (1 - Z_i)\,S\right)\left(\sum_{k=N_v+1}^{N} O_k\right) + \left(\sum_{i=N_v+1}^{N} O_i\right)\left(\sum_{k=1}^{N_v} Z_k O_k + (1 - Z_k)\,S\right)\\ &+ \left(\sum_{i=N_v+1}^{N} O_i\right)\left(\sum_{k=N_v+1}^{N} O_k\right)\Bigg].\end{aligned} \tag{S6}$$

Again applying the distributive law, we get terms where $i = k$ and terms where $i \neq k$:

$$\begin{aligned}\mathrm{E}[X^2] = \frac{1}{N^2}\,\mathrm{E}\Bigg[&\sum_{i=1}^{N_v} (Z_i O_i + (1 - Z_i)\,S)^2 + \sum_{i=1}^{N_v}\sum_{k\neq i}^{N_v} (Z_i O_i + (1 - Z_i)\,S)\,(Z_k O_k + (1 - Z_k)\,S)\\ &+ \sum_{i=1}^{N_v}\sum_{k=N_v+1}^{N} Z_i O_i O_k + \sum_{i=1}^{N_v}\sum_{k=N_v+1}^{N} (1 - Z_i)\,S O_k + \sum_{i=N_v+1}^{N}\sum_{k=1}^{N_v} Z_k O_k O_i\\ &+ \sum_{i=N_v+1}^{N}\sum_{k=1}^{N_v} (1 - Z_k)\,S O_i + \sum_{i=N_v+1}^{N} {O_i}^2 + \sum_{i=N_v+1}^{N}\sum_{k\neq i}^{N} O_i O_k\Bigg],\end{aligned} \tag{S7}$$

which further simplifies to

$$\begin{aligned}\mathrm{E}[X^2] = \frac{1}{N^2}\Bigg[&N_v\left(D\left(\mu_o^2 + \sigma_o^2\right) + (1 - D)\left(\mu_s^2 + \sigma_s^2\right)\right)\\ &+ N_v\,(N_v - 1)\left(D^2\mu_o^2 + 2D\,(1 - D)\,\mu_s\mu_o + (1 - D)^2\left(\mu_s^2 + \sigma_s^2\right)\right)\\ &+ 2N_v\,(N - N_v)\left(D\mu_o^2 + (1 - D)\,\mu_s\mu_o\right)\\ &+ (N - N_v)\left(\mu_o^2 + \sigma_o^2\right) + (N - N_v)(N - N_v - 1)\,\mu_o^2\Bigg].\end{aligned} \tag{S8}$$

The second term of Eqn. S3 expands to

$$\mathrm{E}[XS] = \frac{1}{N}\,\mathrm{E}\left[Z_i O_i S + (1 - Z_i)\, S^2 + O_i S\right], \tag{S9}$$

$$\mathrm{E}[XS] = \frac{1}{N}\left[N_v\left(D\mu_o\mu_s + (1 - D)\left(\mu_s^2 + \sigma_s^2\right)\right) + (N - N_v)\,\mu_o\mu_s\right]. \tag{S10}$$

Using the expanded terms in S8 & S10 in S3,

$$\begin{aligned}
\mathrm{MSE} = \frac{1}{N^2}\Bigg[&\mu_o^2\left(N_v D\big(1 + (N_v - 1)\, D + 2\,(N - N_v)\big) + (N - N_v)^2\right)\\
&+ \sigma_o^2\,(N_v D + N - N_v)\\
&+ \left(\mu_s^2 + \sigma_s^2\right)\left(N_v\,(1 - D)\,\big(1 + (N_v - 1)\,(1 - D)\big)\right)\\
&+ \mu_s\mu_o\Big(2N_v\,(N_v - 1)\, D\,(1 - D) + 2N_v\,(N - N_v)\,(1 - D)\Big)\Bigg]\\
&- \frac{2}{N}\left[N_v\Big(D\mu_o\mu_s + (1 - D)\left(\mu_s^2 + \sigma_s^2\right)\Big) + (N - N_v)\,\mu_o\mu_s\right] + \left(\mu_s^2 + \sigma_s^2\right),
\end{aligned} \tag{S11}$$

$$\begin{aligned}
\mathrm{MSE} = \frac{1}{N^2}\Bigg[&\mu_o^2\left(N_v D\,(1 - D) + (N - N_v + N_v D)^2\right)\\
&+ \sigma_o^2\,(N_v D + N - N_v)\\
&+ \left(\mu_s^2 + \sigma_s^2\right)\left(N_v\,(1 - D)\,\big(D + N_v\,(1 - D)\big)\right)\\
&+ 2\mu_s\mu_o\Big(N_v\,(1 - D)\,\big((N_v - 1)\, D + (N - N_v)\big)\Big)\Bigg]\\
&- \frac{2}{N}\left[N_v\Big(D\mu_o\mu_s + (1 - D)\left(\mu_s^2 + \sigma_s^2\right)\Big) + (N - N_v)\,\mu_o\mu_s\right] + \left(\mu_s^2 + \sigma_s^2\right),
\end{aligned} \tag{S12}$$

$$\begin{aligned}
\mathrm{MSE} = \mu_o^2&\left(\frac{N_v D\,(1 - D)}{N^2} + \left(1 - \frac{N_v\,(1 - D)}{N}\right)^2\right)\\
&+ \left(\mu_s^2 + \sigma_s^2\right)\left(\frac{N_v D\,(1 - D)}{N^2} + \left(1 - \frac{N_v\,(1 - D)}{N}\right)^2\right)\\
&+ 2\mu_o\mu_s\left(\frac{N_v\,(1 - D)}{N^2}\Big((N_v - 1)\, D + (N - N_v)\Big) - \frac{N_v D}{N} - \frac{(N - N_v)}{N}\right)\\
&+ \sigma_o^2\left(\frac{(N_v D + N - N_v)}{N^2}\right),
\end{aligned} \tag{S13}$$

$$\text{MSE} = \left[\left(1 - \frac{N_v\left(1-D\right)}{N}\right)^2 + \frac{N_v D\left(1-D\right)}{N^2}\right]\left(\sigma_s^2 + \left(\mu_o - \mu_s\right)^2\right) + \left(\frac{\left(N_v D + N - N_v\right)}{N^2}\right)\sigma_o^2. \tag{S14}$$

Substituting Eqn. S14 in Eqn. S2,

$$V = 1 - \left[\left(1 - \frac{N_v\left(1-D\right)}{N}\right)^2 + \frac{N_v D\left(1-D\right)}{N^2}\right]\left(\sigma_s^2 + \left(\mu_o - \mu_s\right)^2\right) - \left(\frac{\left(N_v D + N - N_v\right)}{N^2}\right)\sigma_o^2. \tag{S15}$$
