# Supplementary material for: Drone swarm strategy for the detection and tracking of occluded targets in complex environments
Source: Commun Eng. 2023 Aug 2;2:55. doi: 10.1038/s44172-023-00104-0 (PMC10956045; doi:10.1038/s44172-023-00104-0)
Supplement: Supplementary file 3 — Description of Additional Supplementary Files [file 44172_2023_104_MOESM3_ESM.pdf]

Description of Additional Supplementary Files

File name: Supplementary Data 1

Description: Simulation plots of all experiments (Target visibility (%) vs. time (seconds))

File name: Supplementary Movie Abstract

Description: Overview

File name: Supplementary Movie 1

Description: Blind sampling strategies (sequentially sampling single camera drone and parallelly sampling camera array)

File name: Supplementary Movie 2

Description: – Enhanced target visibility using particle swarm optimization compared to blind sampling strategies

File name: Supplementary Movie 3

Description: Varying swarm size and forest density.

File name: Supplementary Movie 4

Description: Motion tracking (linear path)

File name: Supplementary Movie 5

Description: Motion tracking (circular path)

File name: Supplementary Movie 6

Description: Failure case: too fast target

File name: Supplementary Movie 7

Description: Failure case : locally too dense occlusion
